# Intranasal ChAdOx1 nCoV-19/AZD1222 vaccination reduces viral shedding after SARS-CoV-2 D614G challenge in preclinical models

**DOI:** 10.1126/scitranslmed.abh0755

**Published:** 2021-08-18

**Authors:** Neeltje van Doremalen, Jyothi N. Purushotham, Jonathan E. Schulz, Myndi G. Holbrook, Trenton Bushmaker, Aaron Carmody, Julia R. Port, Claude K. Yinda, Atsushi Okumura, Greg Saturday, Fatima Amanat, Florian Krammer, Patrick W. Hanley, Brian J. Smith, Jamie Lovaglio, Sarah L. Anzick, Kent Barbian, Craig Martens, Sarah C. Gilbert, Teresa Lambe, Vincent J. Munster

**Affiliations:** 1 Laboratory of Virology, National Institute of Allergy and Infectious Diseases, National Institutes of Health, Hamilton, MT 59840, USA.; 2 The Jenner Institute, Nuffield Department of Medicine, University of Oxford, Oxford OX3 7DQ, UK.; 3 Research Technologies Branch, Rocky Mountain Laboratories, National Institutes of Health, Hamilton, MT 59840, USA.; 4 Rocky Mountain Veterinary Branch, National Institute of Allergy and Infectious Diseases, National Institutes of Health, Hamilton, MT 59840, USA.; 5 Department of Microbiology, Icahn School of Medicine at Mount Sinai, New York, NY 10029, USA.; 6 Graduate School of Biomedical Sciences, Icahn School of Medicine at Mount Sinai, New York, NY 10029, USA.

## Abstract

ChAdOx1 nCoV-19/AZD1222 is an approved adenovirus-based vaccine for severe acute respiratory syndrome coronavirus 2 (SARS-CoV-2) currently being deployed globally. Previous studies in rhesus macaques revealed that intramuscular vaccination with ChAdOx1 nCoV-19/AZD1222 provided protection against pneumonia but did not reduce shedding of SARS-CoV-2 from the upper respiratory tract. Here, we investigated whether intranasally administered ChAdOx1 nCoV-19 reduces detection of virus in nasal swabs after challenging vaccinated macaques and hamsters with SARS-CoV-2 carrying a D614G mutation in the spike protein. Viral loads in swabs obtained from intranasally vaccinated hamsters were decreased compared to control hamsters, and no viral RNA or infectious virus was found in lung tissue after a direct challenge or after direct contact with infected hamsters. Intranasal vaccination of rhesus macaques resulted in reduced virus concentrations in nasal swabs and a reduction in viral loads in bronchoalveolar lavage and lower respiratory tract tissue. Intranasal vaccination with ChAdOx1 nCoV-19/AZD1222 reduced virus concentrations in nasal swabs in two different SARS-CoV-2 animal models, warranting further investigation as a potential vaccination route for COVID-19 vaccines.

## Intranasal SARS-CoV-2 vaccination

Protection conferred by severe acute respiratory syndrome coronavirus 2 (SARS-CoV-2) vaccines may be influenced by the route of immunization. As SARS-CoV-2 is a respiratory pathogen, vaccinating intranasally may provide enhanced protection within the respiratory tract. To investigate this possibility, van Doremalen *et al.* vaccinated hamsters and rhesus macaques intranasally with ChAdOx1 nCoV-19/AZD1222, an adenovirus-vectored vaccine in clinical use. In both models, intranasal vaccination reduced viral shedding after SARS-CoV-2 challenge relative to control animals. In hamsters, intranasal vaccination generated increased neutralizing antibody titers as compared to intramuscular vaccination, although both routes were effective at reducing viral loads. Together, these data support further investigation into intranasal vaccination with ChAdOx1 nCoV-19/AZD1222.

## Teaser

Intranasal vaccination of hamsters and rhesus macaques with ChAdOx1 nCoV-19 reduces shedding of SARS-CoV-2 after challenge.

## INTRODUCTION

The coronavirus disease 2019 (COVID-19) pandemic, caused by severe acute respiratory syndrome coronavirus 2 (SARS-CoV-2), initiated the rapid development of vaccines based on a wide variety of platforms. Just 17 months later after the release of the first SARS-CoV-2 genome sequence, several vaccines have been approved and phase 3 clinical trial data have been published (*1*–*3*). These data suggest that vaccines based on the spike (S) protein of SARS-CoV-2, which generate a neutralizing antibody response, can reach an efficacy of up to 95%. Furthermore, several vaccines developed by Astrazeneca/Oxford, Bharat Biotech, CanSinoBIO, the Gamaleya Research Institute, Moderna, Pfizer/BioNTech, Sinopharm, Sinovac, and the Vector Institute have now been approved, fully or for emergency use.

In humans, most SARS-CoV-2 infections will present as asymptomatic or mild upper respiratory tract infection but are still accompanied by shedding of virus from the oral and nasal mucosa (*4*). Depending on the study, shedding in asymptomatic infections was of shorter duration but often to similar viral loads initially (*4*). Asymptomatic and presymptomatic shedding has been associated with SARS-CoV-2 transmission (*5*–*7*).

In preclinical nonhuman primate (NHP) challenge experiments, several vaccines were successful at preventing disease and reducing or preventing virus replication in the lower respiratory tract. However, subgenomic and genomic viral RNA was still detected in nasal samples of these NHP experiments, dependent on vaccine dose (*8*–*12*). Subgenomic viral RNA is indicative of replicating virus in the upper respiratory tract. It is currently unclear whether the detection of virus in nasal swabs in NHPs translates directly to transmissibility in humans after infection.

It is possible that vaccination will result in attenuation or prevention of disease, but infection of the upper respiratory tract will occur even after vaccination, possibly resulting in transmission. Currently, most of COVID-19 vaccines in development use an intramuscular (IM) injection, which predominantly produces a systemic immunoglobulin G (IgG) response and a poor mucosal response (*13*). For a vaccine to elicit mucosal immunity, antigens will need to be encountered locally at the initial site of replication: the upper respiratory tract. To address this, we evaluated the potential of using the COVID-19 vaccine candidate, ChAdOx1 nCoV-19, as an intranasal (IN) vaccine in hamster and rhesus macaque models.

## RESULTS

### IN vaccination of Syrian hamsters with ChAdOx1 nCoV-19 protects against SARS-CoV-2 infection after direct challenge

**Fig. 1.**
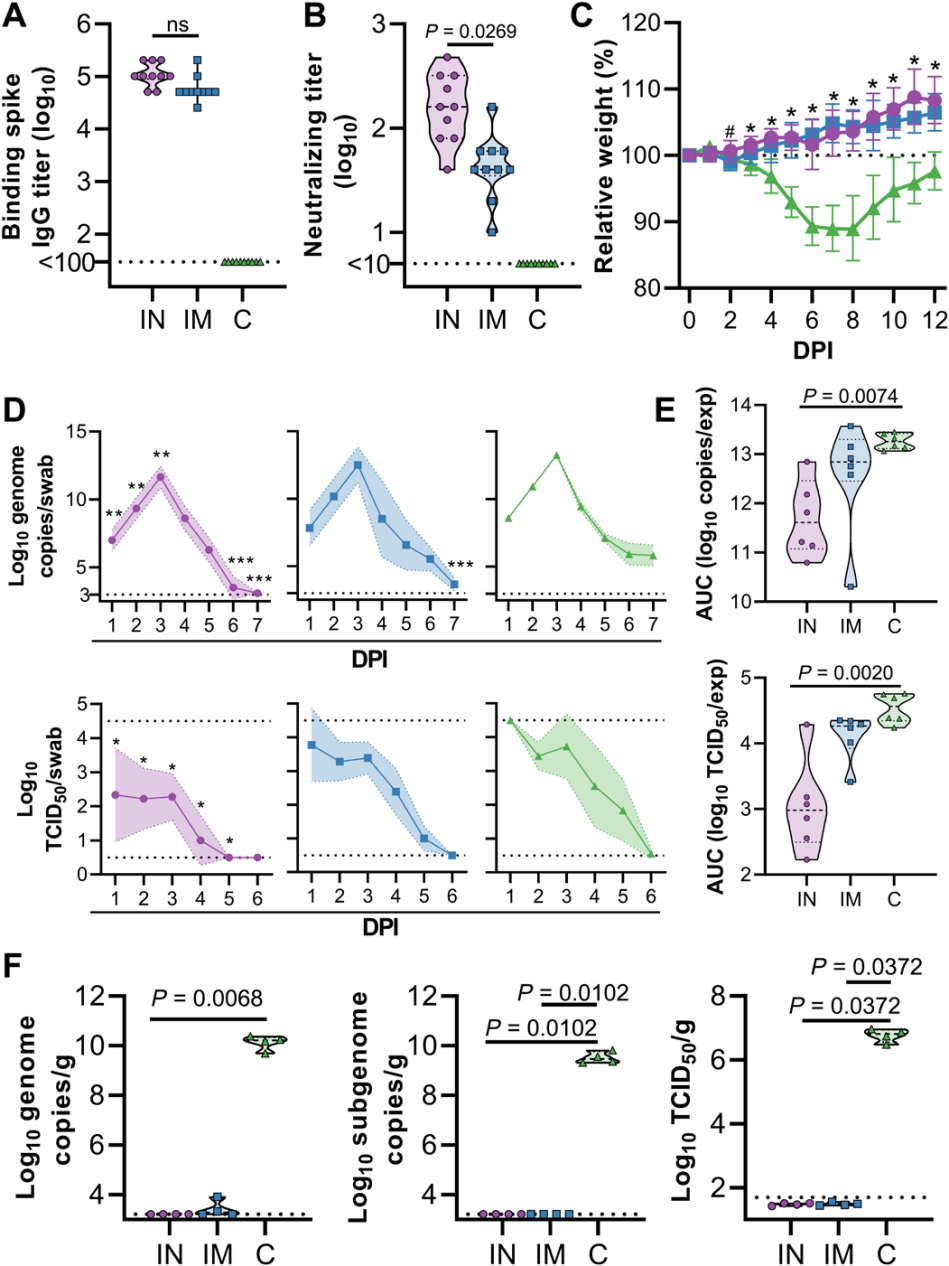
IN ChAdOx1 nCoV-19 vaccination protects Syrian hamsters from SARS-CoV-2 infection. Hamsters (*n* = 10 per group) were vaccinated via the IN route (purple), IM route (blue), or with control (C) vaccine ChAdOx1 GFP via the IM route (green). (**A**) Binding antibody titers against SARS-CoV-2 S protein in serum are shown for day 28 after vaccination. (**B**) VN antibody titers in serum are shown for day 28 after vaccination. For (A) and (B), the geometric mean and 95% confidence interval are shown. Dotted line, limit of detection. (**C**) Relative weight was measured as a percent of starting weight at indicated days postinoculation (DPI) with SARS-CoV-2. #*P* < 0.05 between IN and control group; *P < 0.05 between vaccinated groups and control group. Geometric mean and 95% confidence interval are shown. (**D**) Viral load and viral titer in oropharyngeal swabs are shown as geometric mean (symbols) and 95% confidence interval (shade). *P < 0.05, **P < 0.01, and ***P < 0.001 relative to controls at the same time point. Dotted line, limit of detection. (**E**) Area under the curve (AUC) analysis is shown for viral load and titer detection in oropharyngeal swabs over 7 days postinoculation. (**F**) Viral load and titer in lung tissue isolated at 5 DPI are shown. For (E) and (F), the dashed line within the violin plots indicate median; dotted lines within the violin plot indicate quartiles. Statistical analyses are done using mixed-effect analyses (C), two-way ANOVA (D), or Kruskal-Wallis test (E and F).

To evaluate the efficacy of an IN vaccination with ChAdOx1 nCoV-19, three groups of 10 Syrian hamsters (*14*) were vaccinated with a single dose 28 days before challenge; group 1 received ChAdOx1 nCoV-19 via the IN route, group 2 received the same dose of vaccine via the IM route, and group 3 received control vaccine ChAdOx1 green fluorescent protein (GFP) via the IM route. Binding antibodies against SARS-CoV-2 S protein in peripheral blood were measured at 1 day before infection. Vaccination by either route resulted in high IgG titers (25,600 to 204,800), with no difference observed between vaccination routes (Fig. 1A). Likewise, high neutralizing antibodies titers were detectable at 1 day before infection. Neutralizing antibody titers were significantly higher in animals that received an IN vaccination (*P* = 0.0269; Fig. 1B). For IN inoculation of Syrian hamsters [10^4^ median tissue culture infectious dose (TCID_50_) per animal] 28 days after vaccination, we used the SARS-CoV-2/human/USA/RML-7/2020 isolate, which contains the D614G mutation in the S protein. Animals who received ChAdOx1 GFP started losing weight at 3 days post inoculation (DPI) and did not regain weight until 8 DPI. None of the vaccinated animals lost weight throughout the course of the experiment (Fig. 1C). Oropharyngeal swabs were collected daily from six animals per group up to 7 DPI. Viral RNA was detected in swabs from all animals. A reduced amount of viral RNA was detected in nasal swabs from IN-vaccinated animals compared to control animals on 1 to 3 and 6 to 7 DPI (Fig. 1D, top row). However, a significant reduction in viral RNA detected in oropharyngeal swabs from IM-vaccinated animals compared to control animals was only detected at 7 DPI (*P* < 0.05, Fig. 1D, top row). When the area under the curve (AUC) was calculated as a measurement of total amount of viral RNA detected in swabs, values for IN-vaccinated animals were significantly less than control animals (*P* = 0.0074; Fig. 1E, top). Although viral RNA is an important measurement, the most crucial measurement in swabs is infectious virus. We found a significant difference between infectious virus detected in oropharyngeal swabs of IN-vaccinated animals compared to controls daily (*P* < 0.05; Fig. 1D, bottom row). Likewise, the amount of infectious virus detected in swabs over the course of the experiment was significantly lower in IN-vaccinated animals than in controls (*P* = 0.002; Fig. 1E, bottom row). In contrast, we did not find a difference in AUC for viral RNA and infectious virus when comparing control and IM-vaccinated animals (Fig. 1E). At 5 DPI, four animals in each group were euthanized. Viral load and infectious virus titer were high in lung tissue of control animals, whereas we were unable to detect viral RNA or infectious virus in lung tissue from IN-vaccinated animals (Fig. 1F). Two animals in the IM group were weakly positive for genomic RNA but not for subgenomic RNA or infectious virus (Fig. 1F).

**Fig. 2.**
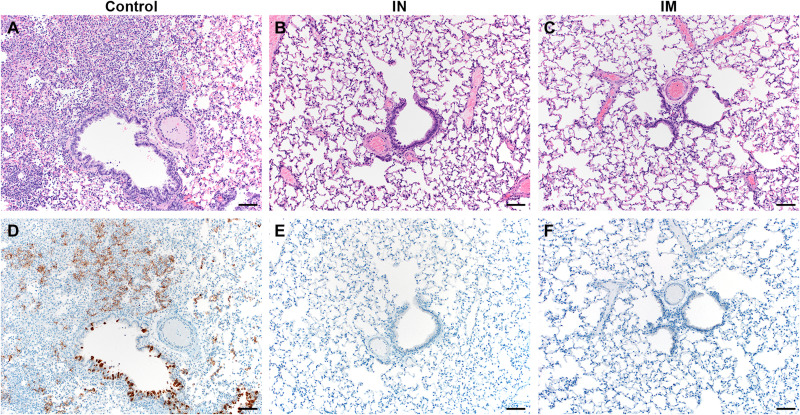
Lung pathology is reduced in ChAdOx1 nCoV-19 IN-vaccinated Syrian hamsters after direct IN challenge with SARS-CoV-2. (**A**) Hematoxylin and eosin (H&E)–stained lung sections from control hamsters 5 days after SARS-CoV-2 infection reveals moderate to marked interstitial pneumonia. (**B** and **C**) H&E-stained sections of lungs isolated from IN-vaccinated (B) or IM-vaccinated (C) hamsters reveal no pathology after infection. (**D**) Numerous immunoreactive (brown) bronchiolar epithelial cells and types I and II pneumocytes are observed in control hamsters. (**E** and **F**) No immunoreactivity is present in sections of lungs isolated from IN-vaccinated (E) or IM-vaccinated (F) hamsters. Scale bars, 50 μm.

Lung tissue obtained at 5 DPI was then evaluated for pathology (Fig. 2). Lesions were found in the lungs of control animals throughout (40 to 70% of tissue; Fig. 2A and table S1). Interstitial pneumonia was present in all animals, as well as edema, type II pneumocyte hyperplasia, and perivascular leukocyte infiltration, similar to what has been observed previously (*14*). In contrast, no lesions or pathology was observed in lung tissue of vaccinated animals (Fig. 2, B and C, and table S1). SARS-CoV-2 N antigen in lung tissue was only found in control animals (20 to 70% of lung tissue was immunoreactive; Fig. 2D) but not for vaccinated animals (Fig. 2, E and F, and table S1).

### IN vaccination of Syrian hamsters with ChAdOx1 nCoV-19 protects against SARS-CoV-2 infection during direct contact with infected hamsters

**Fig. 3.**
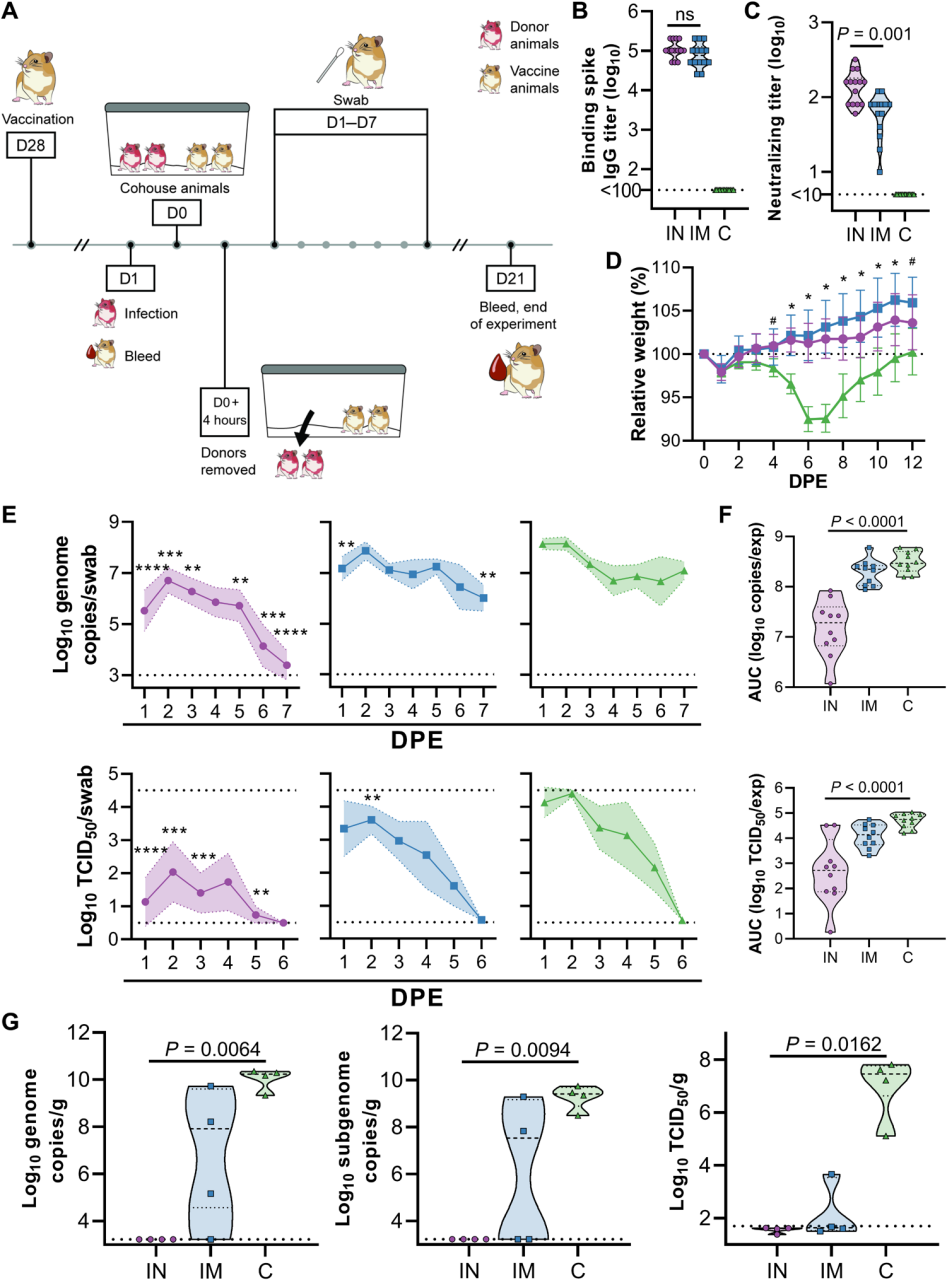
IN ChAdOx1 nCoV-19 vaccination protects Syrian hamsters from SARS-CoV-2 infection by transmission. Hamsters (*n* = 14 per group) were vaccinated via the IN route (purple), IM route (blue), or with control (C) vaccine ChAdOx1 GFP via the IM route (green). (**A**) Hamsters received a single vaccination 28 days before exposure. Donor animals were challenged at −1 days postexposure (DPE), and hamsters were cohoused for 4 hours, 1 day later. (**B**) Binding antibody titers against SARS-CoV-2 S protein in serum are shown for day 28 after vaccination. ns, not significant. (**C**) VN antibody titers in serum are shown for day 28 after vaccination. For (B) and (C), the geometric mean and 95% confidence interval are shown. Dotted line, limit of detection. (**D**) Relative weight was measured as a percent of starting weight at indicated DPE with SARS-CoV-2. #*P* < 0.05 between IN and control group; *P < 0.05 between vaccinated groups and control group. Geometric mean and 95% confidence interval are shown. (**E**) Viral load and viral titers in oropharyngeal swabs are shown as geometric mean (symbols) and 95% confidence interval (shade). **P < 0.01, and ***P < 0.001, relative to controls at the same time point. Dotted line, limit of detection. (**F**) AUC analysis is shown for viral load and titer detection in oropharyngeal swabs over 7 DPE. (**G**) Viral load and titer in lung tissue isolated at 5 DPI are shown. For (F) and (G), the dashed line within the violin plots indicate median; dotted lines within the violin plot indicate quartiles. Statistical analyses done using mixed-effect analyses (D), two-way ANOVA (E), or Kruskal-Wallis test (F and G).

Since direct IN inoculation of Syrian hamsters is an artificial route of virus challenge, and Syrian hamsters transmit SARS-CoV-2 readily (*15*), we repeated the above experiment within a direct contact horizontal transmission setting. Briefly, unvaccinated hamsters were IN challenged with SARS-CoV-2 (10^4^ TCID_50_, donor animals). After 24 hours, vaccinated animals were introduced into the cage. Four hours later, donor animals were removed (Fig. 3A). As in the previous experiment, vaccination of hamsters with ChAdOx1 nCoV-19 resulted in high binding and neutralizing antibodies (Fig. 3, B and C). Neutralizing antibodies were significantly higher in IN-vaccinated animals relative to IM-vaccinated hamsters (*P* = 0.001; Fig. 3C). Control animals started losing weight at 4 days postexposure (DPE) and started recovering weight at 8 DPE (Fig. 3D). None of the vaccinated animals lost weight throughout the experiment, and a significant difference in weight was observed starting at 4 and 5 DPE for IN- and IM-vaccinated animals compared to controls, respectively (*P* < 0.05; Fig. 3D). Oropharyngeal swabs were collected daily from 10 animals per group, and viral RNA and infectious virus was measured (Fig. 3E). A significantly reduced amount of viral RNA and infectious virus was again detected in IN-vaccinated animals relative to control animals (*P* < 0.05; Fig. 3F). However, as in the previous experiment, limited differences in amount of viral RNA and infectious virus were detected in IM-vaccinated animals compared to controls (Fig. 3E). The total amount of viral RNA and infectious virus throughout the experiment, illustrated as AUC, was significantly different for IN-vaccinated animals compared to controls in both viral RNA and infectious virus (*P* < 0.0001) but not for IM-vaccinated animals (Fig. 3F). Four animals per group were euthanized at 5 DPE and lung tissue was harvested. Again, no viral RNA or infectious virus was detected in lung tissue obtained from IN-vaccinated animals (Fig. 3G). However, viral RNA could be detected in lung tissue from three [genomic RNA (gRNA)] and two [subgenomic RNA (sgRNA)] IM-vaccinated animals, and infectious virus was detected in lung tissue of one IM-vaccinated animal (Fig. 3G).

Viral RNA obtained from oropharyngeal swabs was sequenced at 2 and 5 DPE. Sequences obtained at 2 DPE from four different animals contained single-nucleotide polymorphisms (SNPs) in the S protein (Table 1). Two SNPs encoded a nonsynonymous mutation: Asp^839^Glu and Lys^1255^Gln. Three swabs were obtained from IN-vaccinated animals, and one swab was obtained from an IM-vaccinated animal. At 5 DPE, three swabs obtained from hamsters, all from the IN group, contained SNPs in the S protein. Two SNPs encoded a nonsynonymous mutation: His^49^Tyr and Ile^434^Val. No identical SNPs in S protein were seen in different swabs.

**Table 1.** SNPs in SARS-CoV-2 S protein sequences obtained from hamster swabs.

**Nucleotide change**	**Amino acid change S**	**Group**	**Number of reads**	**Days**	**Animal**
			**Mutation/total (%)**		
A23911T	Ala^783^Ala	IN	243/260 (93.5)	2 DPE	10
T24079G	Asp^839^Glu	IN	250/391 (63.9)	2 DPE	6
A24253C	Pro^897^Pro	IN	425/641 (66.3)	2 DPE	2
A25325C	Lys^1255^Gln	IM	273/768 (35.5)	2 DPE	20
C21707T	His^49^Tyr	IN	21/35 (60.0)	5 DPE	5
A22862G	Ile^434^Val	IN	88/90 (97.8)	5 DPE	6
C23248T	Phe^562^Phe	IN	126/126 (100)	5 DPE	1

**Fig. 4.**
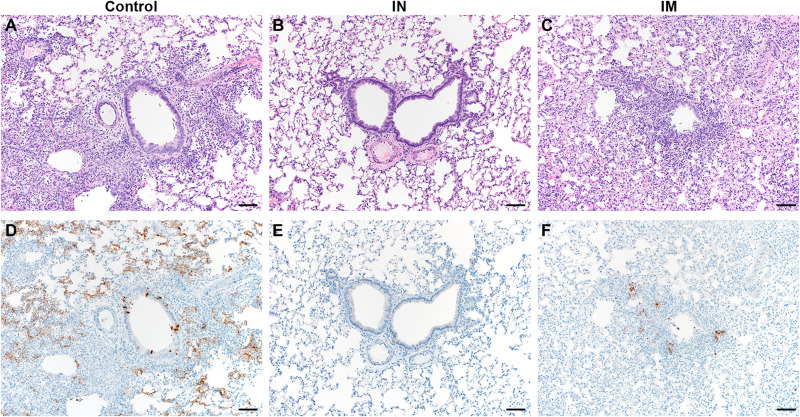
Lung pathology is reduced in ChAdOx1 nCoV-19 IN-vaccinated Syrian hamsters cohoused with SARS-CoV-2 infected hamsters. (**A**) H&E-stained lung sections from control hamsters 5 days after SARS-CoV-2 infection reveal moderate to marked interstitial pneumonia. (**B**) H&E-stained sections of lungs isolated from IN-vaccinated hamsters reveal no pathology after infection. (**C**) H&E-stained sections of lungs isolated from IM-vaccinated hamsters reveal mild interstitial pneumonia after infection. (**D**) Numerous immunoreactive (brown) bronchiolar epithelial cells and types I and II pneumocytes are observed in control hamsters. (**E**) No immunoreactivity is present in sections of lungs isolated from IN-vaccinated. (**F**) Scattered immunoreactive bronchiolar epithelial cells and types I and II pneumocytes are observed in IM-vaccinated hamsters. Scale bars, 50 μm.

Lung tissue of control animals obtained at 5 DPE had the same appearance as those obtained in the previous experiment (Fig. 4A). Lesions were observed in 40 to 50% of tissue, and interstitial pneumonia, edema, type II pneumocyte hyperplasia, and perivascular leukocyte infiltration were observed in all animals. As previously, no lesions or pathology were observed in lung tissue of IN-vaccinated animals (Fig. 4B). However, lesions were observed in the IM-vaccinated animals (5 to 20%, three of four animals), accompanied with mild interstitial pneumonia (three of four animals), type II pneumocyte hyperplasia (two of four animals), and perivascular leukocyte infiltration (one of four animals) (Fig. 4C). Edema was not observed in IM-vaccinated animals (Fig. 4C and table S2). SARS-CoV-2 N antigen in lung tissue was found to be present in control animals (30 to 60% of lung tissue was immunoreactive; Fig. 4D) but not for IN-vaccinated animals (Fig. 4E and table S2). SARS-CoV-2 N antigen in lung tissue was present to a lesser extent in IM-vaccinated animals (5% of lung tissue, three of four animals; Fig. 4F).

### IN vaccination of rhesus macaques with ChAdOx1 nCoV-19 results in a robust immune response

**Fig. 5.**
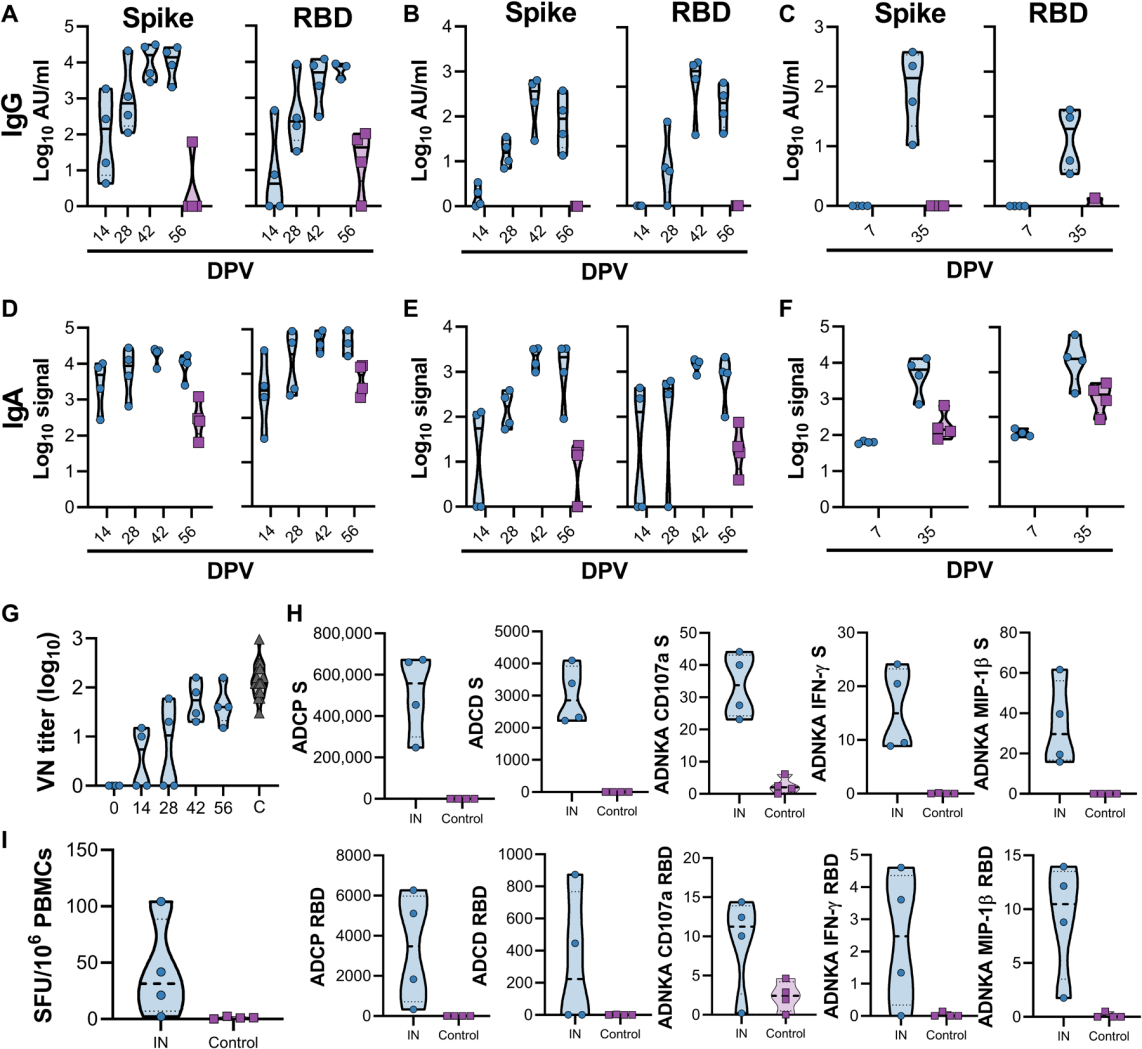
IN vaccination with ChAdOx1 nCoV-19 in rhesus macaques induces humoral and cellular immune responses. (**A** to **C**) Truncated violin plot of SARS-CoV-2–specific IgG antibodies measured in serum (A), nasosorption samples (B), and BAL (C) shows evidence of S- and RBD-specific IgG in all three tissues at the indicated days postprime vaccination (DPV) in animals receiving IN ChAdOx1 nCoV-19 (blue, *n* = 4) but no serum IgG in control animals (purple, *n* = 4). (**D** to **F**) Truncated violin plot of SARS-CoV-2–specific IgA antibodies measured in serum (D), nasosorption samples (E), and BAL (F) shows induction of IgA at all three sites in IN-vaccinated animals. (**G**) Truncated violin plot of neutralizing antibodies in serum is shown compared to convalescent sera (C, black) from individuals with COVID-19. The red triangle indicates NIBSC serum control 20/130. (**H**) Truncated violin plot of effector functions of antibodies in serum are shown. Antibody-dependent NK cell activation (ADNKA) is shown based on expression of CD107a, IFN-γ, and MIP-1β. ADCP, antibody-dependent cellular phagocytosis; ADCD, antibody-dependent complement deposition. (**I**) Truncated violin plots of S protein–specific T cell responses in PBMCs isolated from vaccinated or controls animals at −14 DPI minus −56 DPI response. SFU, spot-forming units. Black lines indicate median; dotted lines indicate quartiles. Blue indicates vaccinated animals and purple indicates control animals (only 56 DPV values are shown).

The results obtained in hamster studies prompted us to investigate the impact of IN vaccination in rhesus macaques (*16*). Four NHPs were vaccinated with a prime-boost regimen of ChAdOx1 nCoV-19 at 56 and 28 days before challenge using the same dose as previously described (*8*), using an IN mucosal atomization device. This device produced a spray of aerosols that were deposited in the nasal cavity. Four control animals were vaccinated with ChAdOx1 GFP as controls. Blood, nasosorption swabs, and bronchoalveolar lavage (BAL) samples were collected throughout the experiment. For nasosorption samples, a small piece of absorbent filter paper was inserted into the nostril of the animal, and the nostril was closed for 60 s. As expected, a higher fraction of IgA to total Ig was detected in nasosorption samples compared to BAL and serum samples (fig. S1). S- and receptor binding domain (RBD)–specific IgG antibodies were detected in serum and nasosorption samples after prime vaccination, but not in BAL, at 7 days postprime vaccination (DPV). Higher IgG titers were found in all samples obtained after a second vaccination at 28 DPV (Fig. 5, A to C). S- and RBD-specific IgA antibodies were detected in serum upon prime vaccination but did not increase upon boost vaccination (Fig. 5D). In contrast, SARS-CoV-2–specific IgA antibodies were only weakly detected in nasosorption samples upon prime vaccination but further increased upon boost vaccination (Fig. 5E). No SARS-CoV-2–specific IgA antibodies were detected in BAL at 7 DPV but were detected 7 days after boost vaccination (36 DPV; Fig. 5F). Circulating neutralizing antibodies were readily detected in vaccinated animals, to values similar to convalescent serum obtained from humans infected with SARS-CoV-2 with symptoms varying from asymptomatic to severe (Fig. 5G) and from serum collected from NHPs that received a prime or prime-boost IM vaccination with ChAdOx1 nCoV-19 28 days after vaccination (*8*). Furthermore, multiple antigen-specific antibody Fc effector functions were detected in serum samples collected at 14, 28, 42, and 56 DPV; circulating antibodies in vaccinated animals promoted phagocytosis, complement deposition, and natural killer (NK) cell activation as measured using in vitro assays (Fig. 5H). S protein–specific T cell responses were detected in peripheral blood mononuclear cells (PBMCs) that were isolated at 14 DPV compared to control animals (Fig. 5I).

### IN vaccination of rhesus macaques with ChAdOx1 nCoV-19 protects against SARS-CoV-2 infection

**Fig. 6.**
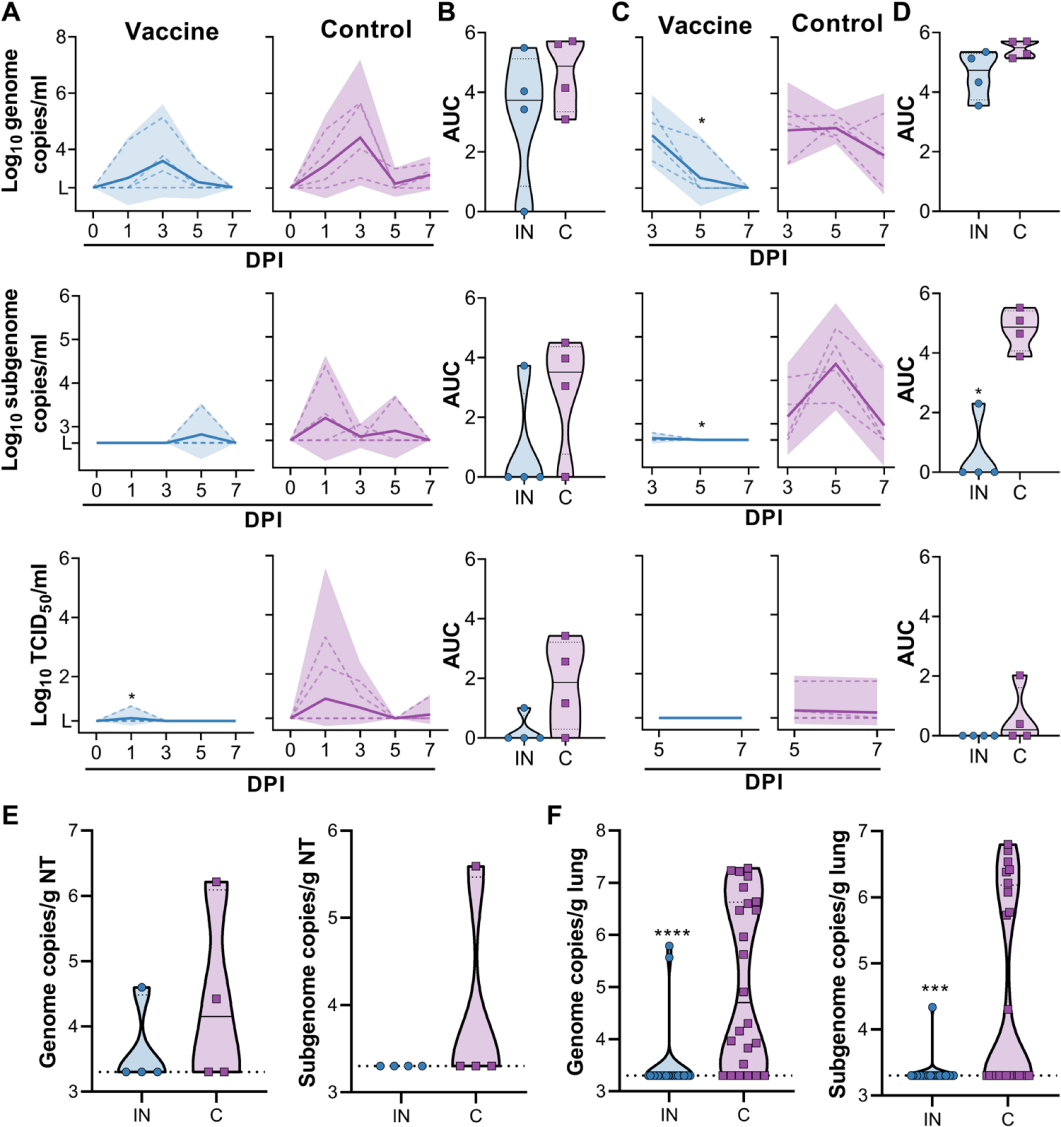
SARS-CoV-2 detection in samples obtained from rhesus macaques upon virus challenge. (**A**) gRNA, sgRNA, and infectious virus concentrations were measured in nasal swabs at indicated days postinoculation (DPI). (**B**) AUC was calculated as an indication of the total amount of virus shed in nasal swabs. (**C**) gRNA, sgRNA, and infectious virus concentrations were measured in BAL. (**D**) AUC was calculated as an indication of the total amount of virus shed in BAL. (**E** and **F**) Amount of gRNA and sgRNA in nasal turbinates (E) and lung tissue (F) is shown. For all panels, blue indicates vaccinated animals and purple indicates control animals. For (A) and (C), dotted lines indicate individual animals and solid lines indicate geometric mean; shaded areas indicate 95% confidence intervals. For (B) and (D) to (F), solid lines indicate median and dotted lines indicate quartiles. *P < 0.05, ***P < 0.001, and ****P < 0.0001, as determined by two-tailed Mann-Whitney test.

Animals were challenged via the intratracheal and IN route using 10^6^ TCID_50_ of SARS-CoV-2 (SARS-CoV-2/human/USA/RML-7/2020). Nasal swabs were investigated for the presence of genomic RNA, subgenomic RNA, and infectious virus. In control animals, both types of viral RNA were readily detected in nasal swabs (Fig. 6A). Genomic RNA was detected in all four animals (11 of 16 swabs total), whereas subgenomic RNA was detected in three of four animals (4 of 16 swabs total; Fig. 6A). Infectious virus was detected in three of four animals (5 of 16 swabs total; Fig. 6A). Viral RNA was detected in nasal swabs obtained from vaccinated animals, but viral load was lower and fewer swabs were positive. Genomic RNA was detected in three of four animals (5 of 16 swabs total), whereas subgenomic RNA and infectious virus was only detected in one of four animals (one swab each) (Fig. 6A). Total amount shed was depicted using AUC analysis (Fig. 6B). Genomic and subgenomic RNA in BAL was detected in all four control animals (11 and 8 of 12 samples, respectively; Fig. 6C). Infectious virus in BAL was detected in two of four animals (three of eight samples; Fig. 6C). Genomic RNA was detected in four of four vaccinated animals, but only at early time points (5 of 12 samples; Fig. 6C). Subgenomic RNA was only found in one animal and was very low (1 of 12 samples; Fig. 6C). The differences in number of positive samples between vaccinated and control animals were significant (genomic RNA, *P* = 0.0272; subgenomic RNA, *P* = 0.0094; Fig. 6C). No infectious virus could be detected in BAL samples from vaccinated animals (0 of 12 samples) (Fig. 6C). AUC analyses again showed a significant reduction in sgRNA concentration in BAL from vaccinated animals relative to controls (*P* = 0.0286; Fig. 6D). Animals were euthanized at 7 DPI, and viral RNA concentrations in nasal turbinates and lung tissue were analyzed. Viral load in lung was significantly lower for vaccinated animals than for control animals (*P* < 0.0001 and 0.001 for genomic and subgenomic RNA, respectively), but no difference in viral load in nasal turbinates was detected (Fig. 6, E to F).

**Fig. 7.**
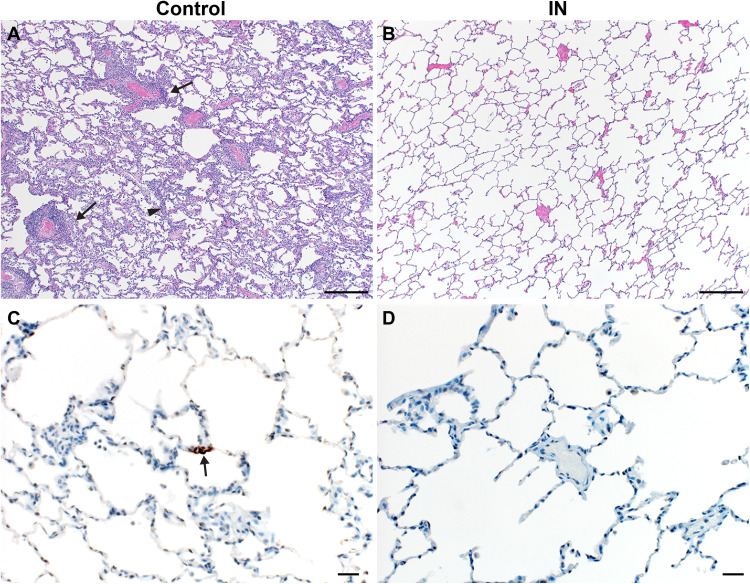
Lung pathology is reduced in ChAdOx1 nCoV-19 IN-vaccinated rhesus macaques after SARS-CoV-2 challenge. (**A** and **B**) Lung tissue sections isolated from IN-vaccinated (A) and control (B) rhesus macaques were stained with H&E. Scale bars, 200 μm. (A) Interstitial pneumonia (arrowhead) and lymphocytic perivascular cuffing (arrows) were observed in control samples. (B) No pathology was observed in IN-vaccinated lung samples. (**C** and **D**) Immunohistochemistry for SARS-CoV-2 antigen (brown) reveals rare type I pneumocyte immunoreactivity (arrow) in control samples (C) but no immunoreactivity in IN-vaccinated samples (D). Scale bars, 20 μm.

Three of four control animals developed some degree of viral interstitial pneumonia (Fig. 7A). Pulmonary lesions consisted of minimal interstitial pneumonia, characterized by focal areas of type II pneumocyte hyperplasia. These findings are consistent with previously observed responses at 7 DPI with SARS-CoV-2 in rhesus macaques, which is predominantly a reparative response. In addition, a mild thickening of alveolar septa and small numbers of macrophages and fewer neutrophils were observed. Multifocally, there were perivascular infiltrates of small numbers of lymphocytes that form perivascular cuffs. Pulmonary pathology was absent in lung tissue of vaccinated animals after inoculation with SARS-CoV-2 (Fig. 7B). Immunohistochemistry analysis showed that no SARS-CoV-2 antigen was detected by immunohistochemistry in vaccinated animals (Fig. 7C). In contrast, viral antigen was observed in types I and II pneumocytes in all control animals (Fig. 7D).

**Fig. 8.**
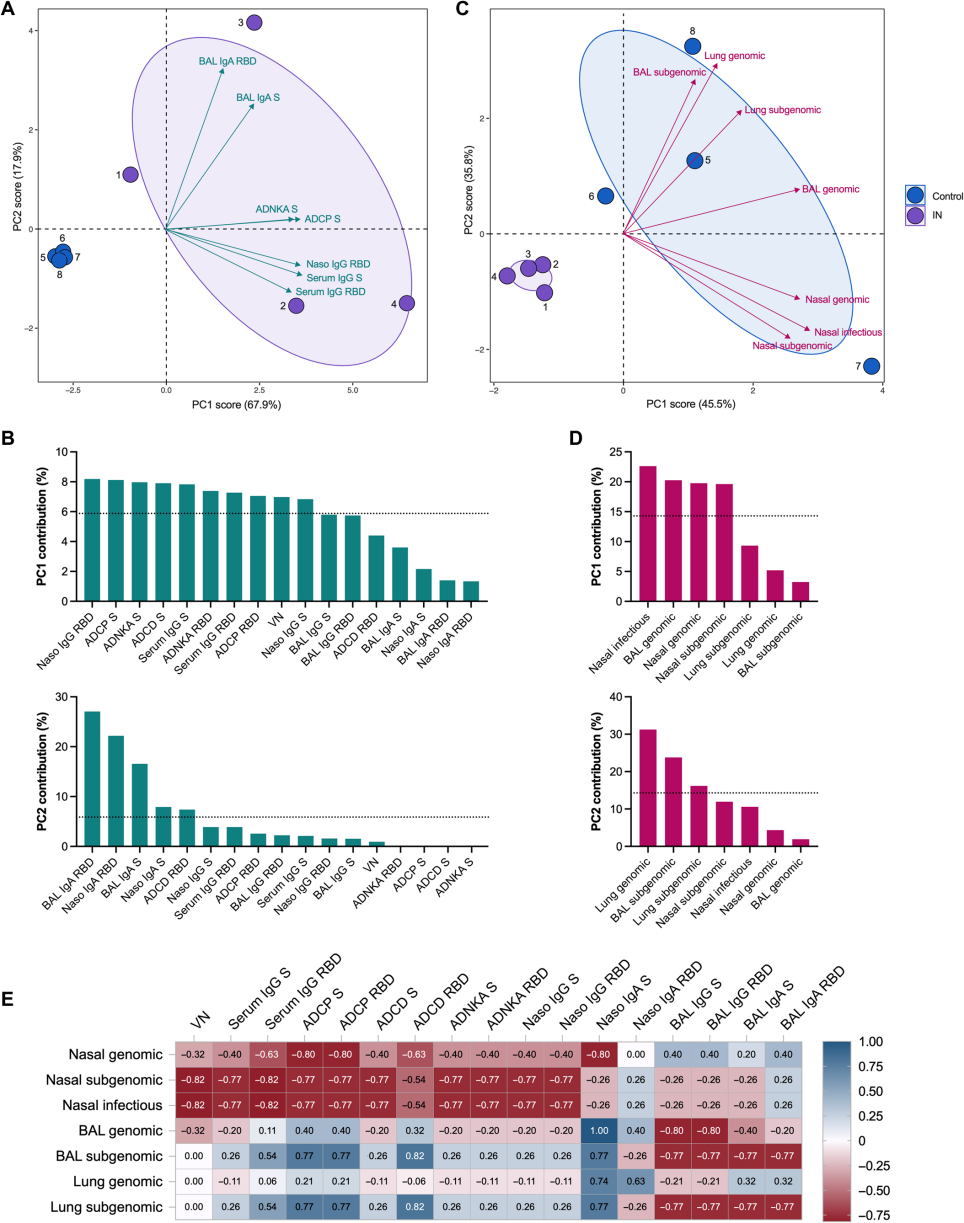
Vaccine-induced humoral responses influence viral RNA titers after SARS-CoV-2 challenge. (**A**) Principal components analysis (PCA) plot of the multivariate antibody profile across all animals (numbered dots) is shown. Ellipses indicate group distribution as 95% confidence interval. Mapped arrow projections indicate the influence of individual variables on the principal components (PCs); the plot depicts only the top seven contributors. (**B**) The complete antibody variable loading plots for PC1 and PC2 are shown with a dotted line to indicate average expected contribution. (**C**) PCA plot of the multivariate AUC virology profile across all animals (numbered dots) is shown. Ellipses indicate group distribution as 95% confidence interval. Mapped arrow projections indicate the influence of individual variables on the PCs. (**D**) The complete virology variable loading plots for PCs 1 and 2 are shown with a dotted line to indicate average expected contribution. (**E**) A heatmap visualization is shown of the correlations between antibody measures and viral RNA (AUC) titers for the IN-vaccinated animals. *R* values were generated using two-sided Spearman rank correlation tests. Naso, nasosorption samples; VN, virus neutralization titer.

We subsequently sought to define the impact of the vaccine-specific humoral response on nasal shedding and viral load after challenge. We first used a principal components analysis (PCA) to cumulatively assess and identify salient drivers of variance across multivariate antibody responses, in particular within the cohort of ChAdOx1 nCoV-19 recipients. PCA revealed the distinct segregation of vaccinated animals from controls, driven by local and systemic antibodies with diverse functions (Fig. 8, A and B). Variation between IN-immunized animals, largely encapsulated by principal component (PC) 2, was primarily mediated by differences in virus-specific IgA or IgG antibody titers in BAL and nasosorption samples. Minimal titers of nasosorption IgG and relatively low titers of nasosorption IgA were detected in the only animal exhibiting virus titer in nasal swabs after challenge (NHP1). This animal also had low serum IgG and virus-neutralizing (VN) titers. Meanwhile, titers of BAL IgA and IgG were lowest in NHP2 and very high in NHP4; genomic and subgenomic RNA titers in BAL and lung tissue were highest and lowest in these animals, respectively. PCA of postchallenge viral load (AUC) in nasal swabs, BAL, and lung tissue, again, yielded clustering according to vaccination status (Fig. 8C). Variation between control animals seemed to reflect site-specific differences in virus replication between the upper and lower respiratory tract (Fig. 8D).

To examine these relationships further, we generated a correlation matrix integrating the pre- and postchallenge data from the IN-vaccinated animals (fig. S2). The Spearman rank correlation coefficients computed for individual antibody-virology variable pairings were assessed; however, the low number of animals precluded statistical comparison. Nevertheless, higher titers of serum (neutralizing and Fc effector function–inducing) antibodies and nasosorption antibodies correlated with reduced virus detection in nasal swabs, and viral RNA in the BAL and lung tissue exclusively displayed strong negative correlations with BAL IgG and IgA titers (Fig. 8E). Subgenomic RNA titers generally appeared to correlate more strongly with antibody titers than genomic RNA titers across sampling sites.

## DISCUSSION

Here, we show that IN vaccination of hamsters and NHPs with ChAdOx1 nCoV-19 results in a robust mucosal and humoral immune response. In comparison to hamsters vaccinated via the IM route, a reduction in virus load in swabs is found in IN vaccinated animals, combined with full protection of the respiratory tract (no viral RNA). In NHPs, we observed a reduction in infectious virus in swabs at 1 DPI (*P* < 0.05). Viral load in BAL and the lower respiratory tract was reduced, and we were unable to find any signs of pneumonia in vaccinated hamsters or NHPs.

Since the release of the first full-length genome of SARS-CoV-2 (*17*), thousands of complete genomes have been released. Multiple clades have been identified, as well as mutations throughout the genome of SARS-CoV-2. The most prevalent of these mutations is likely D614G in the S protein, which is present in the majority of circulating SARS-CoV-2 viruses (*18*). All vaccines in clinical trials are based on the initial SARS-CoV-2 sequences (*17*), and mutations in the S protein may result in immune evasion (*19*). Here, a heterologous challenge was implemented in all experiments; we used isolate SARS-CoV-2/human/USA/RML-7/2020, which was isolated from a nasopharyngeal swab in July 2020 and belongs to clade 20A. This virus has only the D614G mutation as compared to the vaccine antigen. Both hamster and NHP studies described here demonstrated that the ChAdOx1 nCoV-19 vaccine protects against SARS-CoV-2 containing the D614G mutation. It is likely that this translates to other vaccine platforms as well.

Our previous and others’ studies investigating efficacy of COVID-19 vaccines in NHPs showed complete or near-complete protection of the lower respiratory tract, but virus detection in nasal swabs was still observed (*8*–*12*). In natural infection with respiratory pathogens, a systemic immune response, dominated by IgG, as well as a mucosal immune response, dominated by secretory IgA (sIgA), is induced (*13*, *20*). Although abundant literature exists on systemic immune responses to natural SARS-CoV-2 infection, literature on mucosal immunity is currently limited. In mucosal fluids from patients with COVID-19, S- and RBD-specific IgA, IgG, and IgM were readily detected (*21*–*23*). It is hypothesized that sIgA mainly protects the upper respiratory tract, whereas systemic IgG protects the lower respiratory tract (*13*, *24*, *25*).

Upon IN vaccination of rhesus macaques with ChAdOx1 nCoV-19, we were able to detect SARS-CoV-2–specific IgG and IgA in serum. SARS-CoV-2–specific IgG and IgA were also detected in nasosorption samples and BAL. No nasosorption samples were collected in our previous study (*8*), but BAL collected at 3 and 5 DPI did not contain high titers of SARS-CoV-2–specific antibodies. Thus, IN vaccination elicited enhanced SARS-CoV-2–specific mucosal immunity comparable to that induced in animals who received an IM vaccination with ChAdOx1 nCoV-19 as demonstrated by IgA detection in nasosorption and BAL samples. Furthermore, in NHPs, subgenomic and infectious virus in swabs was only detected in one vaccinated animal. This animal exhibited low titers of IgG and IgA antibodies in nasosorption samples coupled with low VN and sera IgG titers, suggesting that a robust humoral response in the nasal mucosa and in circulation is necessary to efficiently control nasal shedding.

T cell responses may contribute to vaccine-mediated protection against SARS-CoV-2. S protein–specific T cell responses were detected in three of four vaccinated animals and were comparable to T cell responses detected in rhesus macaques that received a prime-boost regimen of ChAdOx1 nCoV-19 via the IM route as measured by enzyme-linked immune absorbent spot (ELIspot) assay.

Vaccination of small-animal models with an adeno-vectored vaccine against SARS-CoV-2 has been reported by others, including two studies which investigated IN vaccination (*26*–*28*). Bricker *et al.* (*28*) showed a reduction in virus load in nasal swabs, near complete protection of upper respiratory tract, and partial lower respiratory tract protection in hamsters, whereas Hassan *et al.* (*26*) did not investigate nasal shedding but found near complete protection of upper and lower respiratory tract tissue in mice. Tostanoski *et al.* (*27*) investigated IM vaccination in hamsters and found near complete protection in lung tissue dependent on vaccine candidate. This agrees with our findings; we find a reduction in virus load in swabs of IN-vaccinated animals but not of IM-vaccinated animals. We also find full protection of the lower respiratory tract in IN-vaccinated animals. Because IN vaccination of mice (*26*) and NHPs elicited SARS-CoV-2–specific IgA in BAL or nasosorption samples, we hypothesize that the same occurred in hamsters and, combined with the higher neutralizing titers, resulted in a reduction in nasal shedding.

In our second hamster study, we moved away from IN inoculation and investigated vaccine efficacy in a transmission model. Transmission of SARS-CoV-2 was efficient, resulting in 100% transmission to control sentinel animals after just 4 hours of exposure to infected animals. Again, IN vaccination resulted in a reduction in virus detection in swabs from sentinel hamsters compared to control animals. Although protection of the lower respiratory tract was complete in IN-vaccinated animals, only partial control was seen in IM-vaccinated animals, in contrast to the direct challenge experiment. It is possible that the difference between IN- and IM-vaccinated animals is caused by virus seeding of the lungs from the upper respiratory tract; higher viral nasal detection in IM-vaccinated animals compared to IN-vaccinated animals is likely reflective of a relative increase in virus deposition in the lung from the upper respiratory tract in IM compared to IN-vaccinated animals. That does not explain however why such a discrepancy between vaccination groups was not observed in the direct challenge study. Another hypothesis would be a difference in the initial site of virus deposition. Direct contact transmission likely represents a wide variety of exposure routes for the sentinel animals, including fomites and aerosols. A previous study in our laboratory showed the deposition of fluorescently labeled virus in the lungs of hamsters upon IN inoculation (*29*). However, whereas that study used an inoculation volume of 80 μl, in the current study, an inoculation volume of 40 μl was used. It is possible that virus deposition directly into the lungs via IN inoculation with 40 μl is limited, whereas in the case of indirect transmission, virus particles might be inhaled directly into the lung. We recently showed that infection via aerosols, but not via direct IN inoculation, resulted in a high virus load in lung tissue at 1 DPI (*30*). It should be noted that infectious virus titers in the lungs of control animals in the transmission experiment compared to the direct challenge experiment were five times higher, supporting this supposition. Last, it is possible that IN vaccination results in a faster and more robust systemic immune response, as demonstrated by the higher VN titers in animals vaccinated via the IN route compared to the IM route. Bricker *et al.* (*31*) report a similar difference in VN titers in hamsters vaccinated via the IN and IM route. This could result in a faster clearance of virus from lung tissue. IN-vaccinated control groups were not included in the hamster studies, and thus effects caused by IN inoculation of hamsters independent of the vaccine were not evaluated. However, since volumes used are identical to those used for virus challenge, and no effect was observed in NHPs that received an IN control vaccine, we hypothesize that this is not a cause of the differences observed between the IM and IN vaccinated groups. In addition, previous work with the IN administration of the ChAdOx1 Middle East respiratory syndrome vaccine in a rodent model did not show any difference between IN- or IM-administrated control ChAdOx1 GFP vaccine groups (*32*).

Mercado *et al.* (*12*) previously showed the importance of different effector functions of antibodies in protection against SARS-CoV-2 in rhesus macaques. We adapted their assays and show that upon IN vaccination with ChAdOx1 nCoV-19, a variety of antibody-dependent Fc effector functions are elicited, including monocyte cellular phagocytosis, complement deposition, and NK cell activation. Although the importance of neutralizing antibodies against SARS-CoV-2 has been convincingly demonstrated in rhesus macaques (*33*), the importance of other effector functions remains unknown. ChAdOx1 nCoV-19 has been shown to induce anti-S–neutralizing antibody titers, as well as antibody-dependent neutrophil and monocyte phagocytosis, complement activation, and NK cell activation (*34*). A selective delay or defect in IgG development has been linked to severe and fatal outcomes in human patients (*35*). A recent study in mice demonstrated that in vitro neutralization did not uniformly correlate with in vivo protection and that binding to Fc receptors was of importance, suggesting that antibody effector functions play a pivotal role in protection against SARS-CoV-2 (*36*). Preliminary PC and correlation analyses suggested that, although both vaccine-induced circulating antibodies with neutralizing and nonneutralizing functionality and upper respiratory antibodies play a role in reducing nasal shedding, virus replication in the airway and lung tissue is primarily controlled by antibodies localized to the lower respiratory tract. However, given that low animal numbers prevented us from establishing clear correlations, further studies will be required to define the relative impact of each component of the multifunctional humoral response on measures of protection.

There are several limitations to our study. In our NHP study, animal numbers were limited. Although we observed differences that were very encouraging between intranasally vaccinated and control animals, these were not significant. Furthermore, no IM-vaccinated group was included in the NHP study, which would have allowed for direct comparison of shedding profiles between these groups. There was no IN-vaccine control group in the hamster studies, which would have allowed for direct comparison between vaccinated and control groups dependent on vaccination route. This group was not included on the basis of previous experiences with the ChAdOx1 platform, where no differences were observed in the IN or IM control vaccinated animals (*32*).

The data presented support the investigation of IN delivery of COVID-19 vaccines. With the rollout of COVID-19 vaccines worldwide, it will be crucial to investigate whether the vaccines provide sterilizing immunity or whether vaccinated people are still susceptible to infection of the upper respiratory tract and onward transmission of the virus. The data presented here demonstrate that SARS-CoV-2–specific mucosal immunity is possible after IN vaccination and results in a reduction in virus detection in nasal swabs in hamsters. The University of Oxford has started a phase 1 clinical trial including 54 healthy adults to investigate IN vaccination of human volunteers with ChAdOx1 nCoV-19.

## MATERIALS AND METHODS

### Study design

Study size calculations for the hamster studies were based on in-house data on shedding profiles and were set up to find statistical significance at a drop in shedding of 40% (power = 80% and α = 0.05). Hamsters were sorted by sex and then randomly divided into groups.

The experimental design of the NHP study, including study size calculation, was based on a previously reported study (*8*). Eight Indian origin rhesus macaques (five females and three males) between 4 and 11 years old were sorted by sex, then by weight, and then randomly divided into two groups of four animals. Animal group size was based on initial model development (*16*). Animals were scored daily by the same person who was blinded to study group allocations using a standardized scoring sheet as previously described (*16*).

### In vivo models

The Institutional Animal Care and Use Committee at Rocky Mountain Laboratories provided all animal study approvals, which were conducted in an Association for Assessment and Accreditation of Laboratory Animal Care–accredited facility, following the basic principles and guidelines in the *Guide for the Care and Use of Laboratory Animals* eighth edition, the Animal Welfare Act, U.S. Department of Agriculture, and the U.S. Public Health Service Policy on Humane Care and Use of Laboratory Animals.

Animals were kept in climate-controlled rooms with a fixed light/dark cycle (12 hours/12 hours). Hamsters were cohoused in rodent cages, fed a commercial rodent chow with ad libitum water, and monitored at least once daily. Rhesus macaques were housed in individual primate cages allowing social interactions; fed a commercial monkey chow, treats, and fruit with ad libitum water; and were monitored at least twice daily. Environmental enrichment for rhesus macaques consisted of a variety of human interaction, commercial toys, videos, and music. The Institutional Biosafety Committee (IBC)–approved work with infectious SARS-CoV-2 virus strains under biosafety level 3 (BSL3) and conditions. All sample inactivation was performed according to IBC-approved standard operating procedures for removal of specimens from high containment.

### Generation of ChAdOx1 nCoV-19 vaccine

ChAdOx1 nCoV-19 was designed as previously described (*8*). Briefly, the S protein of SARS-CoV-2 (GenBank, accession no. YP_009724390.1) was codon optimized for expression in human cell lines and synthesized with the tissue plasminogen activator (tPA) leader sequence at the 5′ end by GeneArt Gene Synthesis (Thermo Fisher Scientific). The sequence, encoding SARS-CoV-2 amino acids 2 to 1273 and tPA leader, was cloned into a shuttle plasmid using In-Fusion cloning (Clontech). The shuttle plasmid encodes a modified human cytomegalovirus major immediate early promoter (IE CMV) with tetracycline operator sites, polyadenylation signal from bovine growth hormone. ChAdOx1 nCoV-19 was prepared using Gateway recombination technology (Thermo Fisher Scientific) between this shuttle plasmid and the ChAdOx1 destination DNA bacterial artificial clone (BAC) vector (*37*), resulting in the insertion of the SARS-CoV-2 expression cassette at the E1 locus. The ChAdOx1 adenovirus genome was excised from the BAC using unique Pme I (New England Biolabs) sites, flanking the adenovirus genome sequence. The virus was rescued and propagated in T-Rex 293 human embryonic kidney cells (Invitrogen). Purification was by CsCl gradient ultracentrifugation. Virus titers were determined by hexon immunostaining assay and viral particles calculated on the basis of spectrophotometry (*38*, *39*).

### Vaccination and infection of hamsters

Syrian hamsters (4 to 6 weeks old) were vaccinated with 100 μl of 2.5 × 10^8^ infectious units of vaccine intramuscularly or 50 μl of 2.5 × 10^8^ infectious units of vaccine intranasally. Animals were vaccinated 28 days before challenge or exposure. One day before virus challenge or exposure, animals were bled via the retro-orbital plexus. For the direct challenge experiment, 10 animals per group were challenged with 40 μl of 10^4^ TCID_50_ SARS-CoV-2/human/USA/RML-7/2020 (MW127503.1) diluted in sterile Dulbecco’s modified Eagle’s medium (DMEM). In the transmission experiment, 14 unvaccinated donor animals per group were challenged with 40 μl of 10^4^ TCID_50_ SARS-CoV-2/human/USA/RML-7/2020 diluted in sterile DMEM. One day later, 14 vaccinated animals per group were cohoused with donor animals at a 2:2 or 1:1 ratio, separated by sex. Four hours later, donor animals were removed from the cage and euthanized. In each experiment, 50% of animals were male and 50% of animals were female. At 5 DPI, four animals were euthanized, and the remaining animals were followed for 21 days after challenge. Weight was recorded daily, and oropharyngeal swabs were taken daily up to 7 days after inoculation in 1 ml of DMEM supplemented with 2% fetal bovine serum, 1 mM l-glutamine, penicillin (50 U/ml) and streptomycin (50 μg/ml; DMEM2). Upon euthanasia, blood and lung tissue were collected and subsequently analyzed for virology and histology.

### Vaccination and infection of NHPs

The vaccine group was vaccinated with 1 ml of ChAdOx1 nCoV-19 using a MAD Nasal IN Mucosal Atomization Device (Teleflex, USA) at −56 and −28 DPI. Within the control group, two animals were vaccinated via the same route with ChAdOx1 GFP, and two animals were vaccinated with ChAdOx1 GFP in 2 ml using an Omron Mesh nebulizer NE-U100. All vaccinations were done with 2.5 × 10^10^ virus particles per animal diluted in sterile phosphate-buffered saline (PBS). Animals were challenged with SARS-CoV-2/human/USA/RML-7/2020 (MW127503.1) diluted in sterile DMEM on 0 DPI; with administration of 4 ml intratracheally and 1 ml intranasally of 2 × 10^5^ TCID_50_/ml virus suspension. Scoring was based on the evaluation of the following criteria: general appearance and activity, appearance of skin and coat, discharge, respiration, feces and urine output, and appetite. Clinical exams were performed on −56, −49, −42, −28, −21, −14, −7, 0, 1, 3, 5, and 7 DPI. Nasosorption samples and blood were collected at all exam dates. Nasosorption samples were collected as previously described (*40*). Briefly, a nasosorption device (Hunt Developments UK Ltd.) was inserted into the nasal cavity, and the nostril was manually held closed for 60 s. The swab was placed in 300 μl of AB-33 K [PBS containing 1% bovine serum albumin (BSA) and 0.4% Tween 20] and vortexed for 30 s. The swab and liquid were placed on a spin filter (Agilent, 5185-5990) and spun at 16,000 rpm for 20 min. Filtered liquid was aliquoted and stored at −80°C. Nasal swabs were collected on 0, 1, 3, 5, and 7 DPI. BAL was performed on 3, 5, and 7 DPI as previously described. For each procedure, 10 to 30 ml of sterile saline was instilled and the sample was retrieved with manual suction (*41*). Animals were euthanized and necropsy was performed on 7 DPI, and the following tissues were collected: cervical lymph node, mediastinal lymph node, nasal mucosa, trachea, all six lung lobes, right and left bronchus, and spleen.

### Cells and virus

SARS-CoV-2/human/USA/RML-7/2020 (MW127503.1) was obtained from a nasopharyngeal swab obtained on 19 July 2020. Virus propagation was performed in VeroE6 cells in DMEM2. The used virus stock was 100% identical to the initial deposited GenBank sequence, and no contaminants were detected. VeroE6 cells were maintained in DMEM supplemented with 10% fetal bovine serum, 1 mM l-glutamine, penicillin (50 U/ml), and streptomycin (50 μg/ml; DMEM10). VeroE6 cells were provided by R. Baric (University of North Carolina at Chapel Hill). Mycoplasma testing is performed at regular intervals, and no mycoplasma was detected.

### Virus titration

Tissue sections were weighed and homogenized in 750 μl of DMEM. Virus titrations were performed by end point titration in VeroE6 cells, which were inoculated with 10-fold serial dilutions of virus swab medium or tissue homogenates in 96-well plates. Plates were spun down for 1 hour at 1000 rpm. When titrating tissue homogenate, cells were washed with PBS and 100 μl of DMEM2. Cells were incubated at 37°C and 5% CO_2_. Cytopathic effect was read 6 days later.

### Virus neutralization

Sera were heat-inactivated (30 min, 56°C), after which twofold serial dilutions were prepared in DMEM2. One hundred TCID_50_ of SARS-CoV-2 strain nCoV-WA1-2020 (MN985325.1) was added. After 1 hour of incubation at 37°C and 5% CO_2_, the virus:serum mixture was added to VeroE6 cells. Cytopathic effect was scored after 6 days at 37°C and 5% CO_2_ for 6 days. The virus neutralization titer was expressed as the reciprocal value of the highest dilution of the serum that still inhibited virus replication.

### RNA extraction and quantitative reverse-transcription polymerase chain reaction

RNA was extracted from nasal swabs and BAL using a QiaAmp Viral RNA kit (Qiagen) according to the manufacturer’s instructions. Tissue was homogenized and extracted using the RNeasy kit (Qiagen) according to the manufacturer’s instructions. Viral gRNA (*42*)– and sgRNA (*43*)–specific assays were used for the detection of viral RNA. RNA (5 μl) was tested with the Rotor-Gene probe kit (Qiagen) or QuantStudio (Thermo Fisher Scientific) according to instructions of the manufacturer. Dilutions of SARS-CoV-2 standards with known genome copies were run in parallel.

### Expression and purification of SARS-CoV-2 S and RBD

Protein production was performed as described previously (*44*, *45*). Expression plasmids encoding the codon-optimized SARS-CoV-2 full-length S and RBD were obtained from K. Corbett and B. Graham [Vaccine Research Center, National Institutes of Health (NIH)] (*46*) and F. Krammer (Icahn School of Medicine at Mt. Sinai) (*47*). Expression was performed in FreeStyle 293-F cells (Thermo Fisher Scientific) maintained in FreeStyle 293 Expression Medium (Gibco) at 37°C and 8% CO_2_ shaking at 130 rpm. Cultures totaling 500 ml were transfected with polyethylenimine at a density of 1 million cells/ml. Supernatant was harvested 7 days after transfection, clarified by centrifugation, and filtered through a 0.22 μM membrane. The protein was purified using Ni–(nitrilotriacetic acid) NTA immobilized metal-affinity chromatography using Ni Sepharose 6 Fast Flow Resin (GE Lifesciences) or Ni-NTA Agarose (Qiagen) and gravity flow. After elution the protein was buffer exchanged into 10 mM tris (pH 8), 150 mM NaCl buffer (S), or PBS (RBD) and stored at −80°C.

### Enzyme-linked immunosorbent assay

Enzyme-linked immunosorbent assays (ELISAs) were performed as described previously (*8*). Briefly, maxisorp plates (Nunc) were coated overnight at 4°C with 100 ng per well S or RBD protein in PBS. Plates were blocked with 100 μl of casein in PBS (Thermo Fisher Scientific) for 1 hour at room temperature. Serum serially diluted two times in casein in PBS was incubated at room temperature for 1 hour. Antibodies were detected using affinity-purified polyclonal antibody peroxidase-labeled goat–anti-monkey IgG (SeraCare, 074-11-021) in casein followed by 3,3′,5,5′-tetramethylbenzidine 2–component peroxidase substrate (SeraCare, 5120-0047). The reaction was stopped using stop solution (SeraCare, 5150-0021) and read at 450 nm. All wells were washed four times with PBS with 0.1% Tween 20 in between steps. Threshold for positivity was set at three times the optical density value of negative control (serum obtained from NHPs before start of the experiment) or 0.2, whichever was higher.

### Ig subtyping and SARS-CoV-2–specific IgG and IgA quantification

Ig subtyping was performed using the Isotyping Panel 1 Human/NHP Kit on the MESO QuickPlex [MesoScale Discovery (MSD), K15203D] per the manufacturer’s instructions. S and RBD antibodies were determined using a V-PLEX SARS-CoV-2 Panel 2 kit (MSD, K15383U and K15385U) per the manufacturer’s instructions. These kits are validated by MESO QuickPlex. Both dimeric and monomeric IgAs are detected using the kit.

### Antibody-dependent complement deposition

Eleven microliters of Red FluoSpheres NeutrAvidin-Labeled Microspheres (Thermo Fisher Scientific, F8775) was coated with biotinylated S (25 μl at 1 mg/ml) or RBD protein (5 μl at 1 mg/ml) for 2 hours at 37°C, washed twice with PBS, and diluted in 1 ml of PBS. Serum was diluted 10× in RPMI 1640 (Gibco). Ten microliters of beads, 40 μl of RPMI 1640, and 50 μl of diluted sera were mixed and incubated for 2 hours at 37°C. Guinea pig complement (Cedarlane, CL4051) was diluted 25× in gelatin veronal buffer (Boston Bioproducts, IBB-300X) and 100 μl was added to the serum:bead complex and incubated at 37°C for 20 min. The serum:bead complex was then washed twice with 15 mM EDTA and incubated with 50 μl of fluorescein isothiocyanate (FITC)–conjugated anti-C3 antibody (100× dilution in PBS; MP Biomedical, 855385) for 15 min at room temperature in the dark. Serum:bead complexes were washed three times with PBS and analyzed on a BD FACSymphony A5 (BD Biosciences) flow cytometer using a high-throughput sampler within 1 hour of completion of protocol. All samples were run in duplicate. Serum:bead complexes were gated by forward scatter versus side scatter to remove debris, followed by red bead fluorescence gating in the phycoerythrin (PE) channel, and then, the geometric mean fluorescent intensity in the FITC channel was determined using FlowJo 10 (BD Biosciences) software and analyzed in GraphPad Prism version 8.3.0.

### Antibody-dependent monocyte cellular phagocytosis

Beads were prepared as described above. Serum was diluted 100× in RPMI 1640, and 100 μl was mixed with 10 μl beads and incubated at 37°C for 2 hours. Beads were washed once with RPMI 1640. THP-1 cells (American Type Culture Collection, TIB-202) were diluted to 1.25 × 10^5^ cells/ml in RPMI 1640. One hundred microliters was added per sample and incubated at 37°C for 18 hours. Cells were fixed in 10% formalin for 15 min at room temperature in the dark, washed twice with PBS, and ran on a BD FACSymphony A5 (BD Biosciences) flow cytometer using a high-throughput sampler. All samples were run in duplicate as described above.

### Antibody-dependent NK cell activation

NK cell activation was assessed using methods similar to those previously described (*1*, *2*). Briefly, cells were isolated from 30 ml of heparin-treated whole blood collected from a healthy human donor (NIH Institutional Review Board, 01-I-N055) using the RosetteSep Human NK Cell Enrichment Cocktail according to the manufacturer’s instructions (STEMCELL Technologies). NK cells were rested overnight at 37°C in complete RPMI 1640 medium supplemented with 10% fetal bovine serum and interleukin-15 (1 ng/ml; STEMCELL Technologies). Nunc MaxiSorp 96-well ELISA plates (Thermo Fisher Scientific) were coated with SARS-CoV-2 S or RBD antigen (3 μg/ml) for 2 hours at 37°C. Plates were subsequently washed and blocked with a solution of 5% BSA in 1× Dulbecco’s PBS overnight at 4°C. Sera samples collected at −56 and 0 DPI were diluted 1:25 in blocking buffer, plated in duplicates, and incubated on the coated ELISA plates for 2 hours at 37°C. NK cells were resuspended in a staining cocktail containing anti–CD107a-PE-Cy7 antibody (1:100, BioLegend), GolgiStop (1:1500, BD Biosciences), and GolgiPlug (1:1000, BD Biosciences). After removal of sera from the plate, 5.0 × 10^4^ NK cells were added per well and incubated at 37°C for 6 hours. Surface staining was carried out at room temperature for 30 min using anti-CD56 brilliant ultraviolet 737 (1:100, BD Biosciences), anti-CD16 brilliant violet (BV) 510 (1:100, BioLegend), and anti-CD3 BV650 (1:200, BD Biosciences) antibodies before fixation and permeabilization using Cytofix/CytoPerm solution (BD Biosciences) per the manufacturer’s instructions. Intracellular staining was performed using anti–interferon-γ (IFN-γ) peridinin-chlorophyll-protein-Cy5.5 (1:50, BioLegend) and anti–macrophage inflammatory protein–1β (MIP-1β)–PE (1:50, BD Biosciences) antibodies. Data acquisition was performed using BD FACSymphony A5 (BD Biosciences). NK cells were identified by gating on CD3^−^ CD16^+^ CD56^+^ cells.

### ELIspot assay

ELIspot assays were performed as described previously (*8*). PBMCs were plated at a concentration of 300,000 to 500,000 cells per well and were stimulated with four contiguous peptide pools that spanned the length of the SARS-CoV-2 S protein sequence at a concentration of 2 μg/ml per peptide (Mimotopes). The ImmunoSpot Human IFN-γ Single-Color Enzymatic ELISpot Assay Kit was performed according to the manufacturer’s protocol (Cellular Technology). ELISpot plates were inactivated in formalin overnight before removal from BSL4 facilities before reading. Analysis was performed using the CTL ImmunoSpot Analyzer and ImmunoSpot Software (Cellular Technology). Spot-forming units per 1.0 × 10^6^ PBMCs were summed across the four peptide pools for each animal.

### cDNA synthesis

Complementary DNAs (cDNAs) were prepared according to Briese *et al.* (*48*). Briefly, RNA was extracted from hamster swabs and tissues following the QiaAmp Viral RNA extraction protocol (Qiagen), and 11 μl was taken into the SuperScript IV First-Strand cDNA synthesis system (Thermo Fisher Scientific) following the manufacturer’s instructions. After ribonuclease H treatment, second-strand synthesis was performed using Klenow fragment (New England Biolabs) following the manufacturer’s instructions. The resulting double-stranded cDNAs (ds-cDNA) were then purified using Ampure XP bead purification (Beckman Coulter) and eluted into 30 μl of water.

### Sequencing library construction and SARS-CoV2 enrichment

To construct sequencing libraries, 25 μl of ds-cDNA was brought to a final volume of 53 μl in elution buffer (Agilent Technologies) and sheared on a Covaris LE220 (Covaris) to generate an average size of 180 to 220 base pairs (bp). The following settings were used: peak incident power, 450 W; duty factor, 15%; cycles per burst, 1000; and time, 300 s. The Kapa HyperPrep kit was used to prepare libraries from 50 μl of each sheared cDNA sample following modifications of the Kapa HyperPrep kit (version 8.20) and SeqCap EZ HyperCap Workflow (version 2.3) user guides (Roche Sequencing Solutions Inc.). Adapter ligation was performed for 1 hour at 20°C using the Kapa Unique-Dual Indexed Adapters diluted to 1.5 μM concentration (Roche Sequencing Solutions Inc.). After ligation, samples were purified with AmPure XP beads (Beckman Coulter) and subjected to double-sided size selection as specified in the SeqCap EZ HyperCap Workflow User’s Guide. Precapture polymerase chain reaction (PCR) amplification was performed using 12 cycles, followed by purification using AmPure XP beads. Purified libraries were assessed for quality on the Bioanalyzer 2100 using the High-Sensitivity DNA chip assay (Agilent Technologies). Quantification of precapture libraries was performed using the Qubit dsDNA HS Assay kit and the Qubit 3.0 fluorometer following the manufacturer’s instructions (Thermo Fisher Scientific).

The myBaits Expert Virus bait library was used to enrich samples for SARS-CoV-2 according to the myBaits Hybridization Capture for Targeted NGS (version 4.01) protocol. Briefly, libraries were sorted according to estimated genome copies and pooled to create a combined mass of 2 μg for each capture reaction. Depending on estimated genome copies, two to six libraries were pooled for each capture reaction. Capture hybridizations were performed for 16 to 19 hours at 65°C and subjected to 8 to 14 PCR cycles after enrichment. SARS-CoV-2–enriched libraries were purified and quantified using the Kapa Library Quant Universal quantitative PCR mix in accordance with the manufacturer’s instructions. Libraries were diluted to a final working concentration of 1 to 2 nM, titrated to 20 pM, and sequenced as 2 × 150 bp reads on the MiSeq sequencing instrument using the MiSeq Micro kit version 2 (Illumina).

### Next-generation sequencing data analysis

Raw fastq reads were adapter trimmed using Cutadapt version 1.12 (*49*), followed by quality trimming and quality filtering using the FASTX Toolkit (Hannon Lab, CSHL). Reads were paired up and aligned to the SARS-CoV-2 genome from isolate SARS-CoV-2/human/USA/RML-7/2020 (MW127503.1) using Bowtie2 version 2.2.9 (*50*). PCR duplicates were removed using Picard MarkDuplicates version 2.18.7 (Broad Institute). Variant detection was performed using GATK HaplotypeCaller version 4.1.2.0 (*51*) with ploidy set to 2. Raw variant calls were filtered for high confidence variants using bcftools filter (*52*) with parameters quality (QUAL) > 500 and depth (DP) > 20.

### Histology and immunohistochemistry

Necropsies and tissue sampling were performed according to IBC-approved protocols. Lungs were perfused with 10% neutral-buffered formalin and fixed for 8 days. Hereafter, tissue was embedded in paraffin, processed using a VIP 6 Tissue-Tek (Sakura Finetek) tissue processor, and embedded in Ultraffin paraffin polymer (Cancer Diagnostics). Samples were sectioned at 5 μm, deparaffinized in xylene, passed through 100% ethanol, and rehydrated in tap water. Samples were stained with Harris hematoxylin (Cancer Diagnostics, no. SH3777), decolorized with 0.125% HCl/70% ethanol, blued in Pureview PH Blue (Cancer Diagnostics, no. 167020), counterstained with eosin 615 (Cancer Diagnostics, no. 16601), dehydrated, and mounted in Micromount (Leica, no. 3801731), coverslipping medium at room temperature. An in-house–generated SARS-CoV-2 nucleocapsid protein rabbit antibody (GenScript) at a 1:1000 dilution was used to detect specific anti–SARS-CoV-2 immunoreactivity, carried out on a Discovery ULTRA automated staining instrument (Roche Tissue Diagnostics) with a Discovery ChromoMap DAB (Ventana Medical Systems) kit. The tissue slides were examined by a board-certified veterinary anatomic pathologist blinded to study group allocations. Eighteen sections, taken from six different lung lobes, were evaluated for each animal; a representative lesion from each group was selected for figures.

### Statistical analysis

Two-tailed Mann-Whitney tests, two-way analysis of variance (ANOVA), mixed-effect analysis, Fisher test, Spearman rank (two-sided) correlation coefficients, or Kruskall-Wallis analysis was conducted to compare differences between groups using GraphPad Prism version 8.3.0. Statistical tests used are identified in the figure legends. PCA was performed using the R packages “FactoMineR” and “factoextra” to compare antibody and virology profiles. Spearman rank (two-sided) correlation coefficients for pairwise comparisons between all variables were generated using the R “cor” function; the correlation matrix was visualized in R using “ggcorplot.”

## Notes

View/request a protocol for this paper from *Bio-protocol*.
