# Supplementary material for: Intranasal ChAdOx1 nCoV-19/AZD1222 vaccination reduces viral shedding after SARS-CoV-2 D614G challenge in preclinical models
Source: Sci Transl Med. 2021 Jul 27;13(607):eabh0755. doi: 10.1126/scitranslmed.abh0755 (PMC9267380; doi:10.1126/scitranslmed.abh0755)
Supplement: Supplementary file 1 — Figs. S1 and S2 Tables S1 and S2 [file scitranslmed.abh0755_sm.pdf]

Science Translational Medicine
AAAS

Supplementary Materials for

Intranasal ChAdOx1 nCoV-19/AZD1222 vaccination reduces viral shedding after SARS-CoV-2 D614G challenge in preclinical models

Neeltje van Doremalen *et al.*

Corresponding author: Vincent J. Munster, vincent.munster@nih.gov

Sci. Transl. Med. **13**, eabh0755 (2021)
DOI: 10.1126/scitranslmed.abh0755

The PDF file includes:

Figs. S1 and S2
Tables S1 and S2

Other Supplementary Material for this manuscript includes the following:

Data file S1

Supplementary Materials

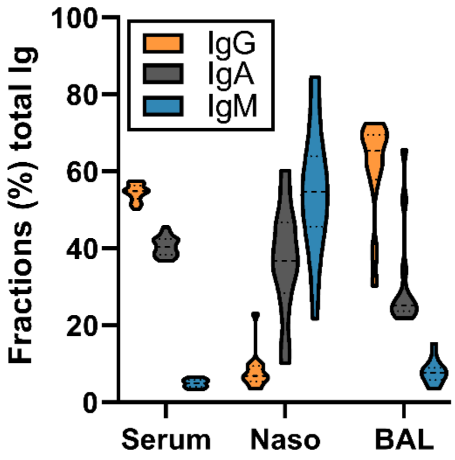

Fig. S1. Fractions of IgA, IgG, and IgM in serum, nasosorption (Naso) or bronchoalveolar lavage (BAL) samples obtained from NHPs at different timepoints from both groups throughout the experiment. Twenty-four serum, 14 nasosorption, and 12 BAL samples were measured by the isotyping panel 1 kit and calculated as a fraction of the total Ig. Data are presented as violin plots showing median and quartiles.

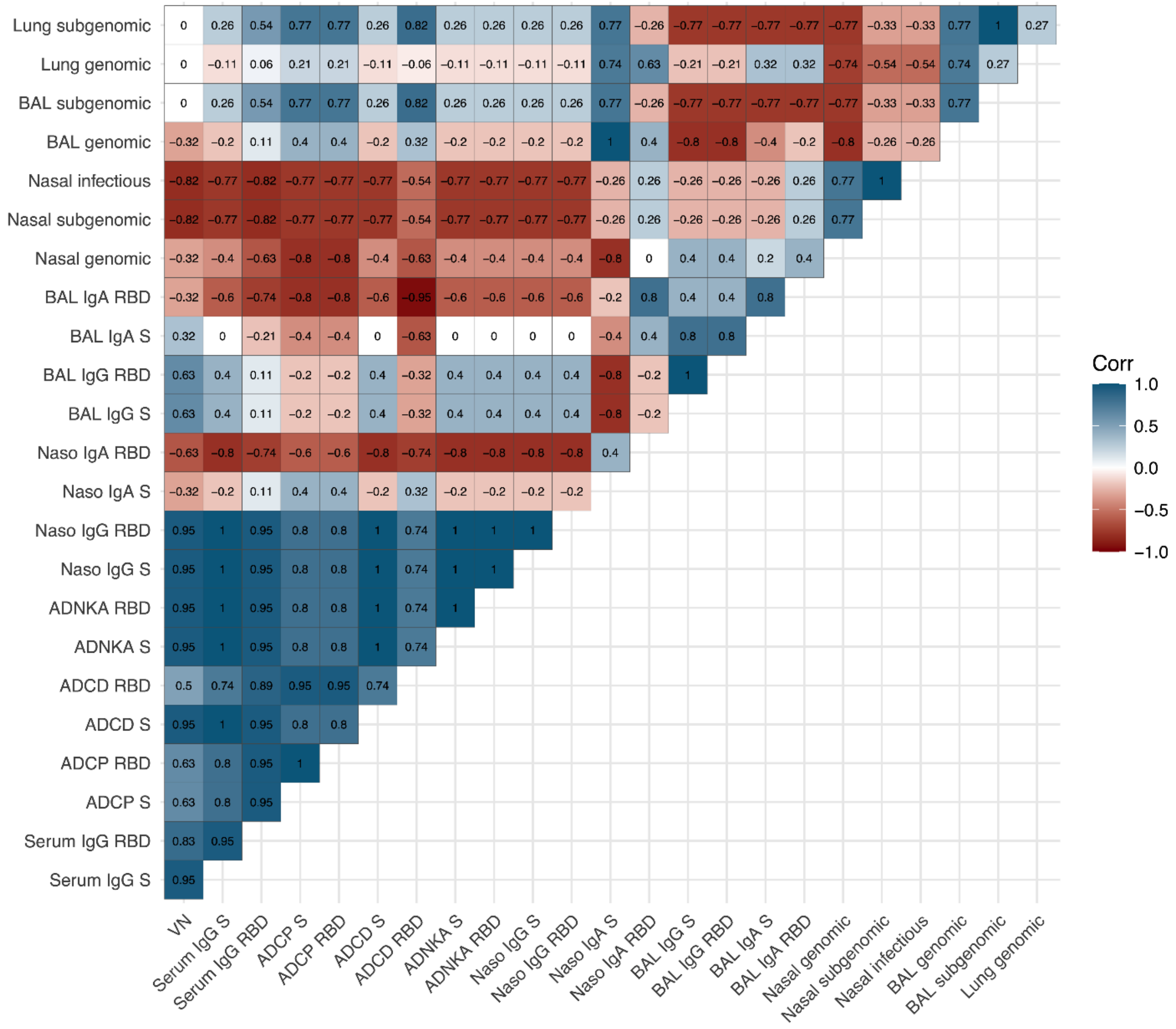

Fig. S2. Correlation matrix featuring all immunology and virology measures. Correlation heatmap, depicted as a matrix, representing pairwise correlations between all antibody and virology variables in IN-vaccinated animals. The two-sided Spearman rank correlation coefficient is indicated within each square. BAL, bronchoalveaolar lavage; S, spike protein; RBD, receptor binding domain; ADNKA, antibody-dependent natural killer cell activation; ADCD, antibody-dependent complement deposition; ADCP, antibody-dependent celullar phagocytosis; VN, virus neutralization.

Table S1. Pathology and immunohistochemistry (IHC) scoring for direct challenge hamster samples. Hematoxylin and Eosin (H&E) stained samples were scored as follows: 0 = not present; 1 = 1-10%; 2 = 11-25%; 3 = 26-50%; 4 = 51-75%; 5 = 76-100%. IHC was scored as follows: 0 = not present; 1 = rare/few; 2 = scattered; 3 = moderate; 4 = numerous; 5 = diffuse. IN, intranasal; IM, intramuscular.

		IN-vaccinated animals				IM-vaccinated animals				Control animals			
H&E	Lesions %	0	0	0	0	0	0	0	0	70	60	40	70
	Interstitial pneumonia	0	0	0	0	0	0	0	0	4	4	3	4
	Bronchiolitis	0	0	0	0	0	0	0	0	2	2	3	3
	Alveolar exudate	0	0	0	0	0	0	0	0	2	2	2	2
	Type II pneumocyte hyperplasia	0	0	0	0	0	0	0	0	2	2	3	2
	Perivascular leukocyte infiltration	0	0	0	0	0	0	0	0	2	2	2	2
	Edema	0	0	0	0	0	0	0	0	2	2	1	3
IHC	Staining %	0	0	0	0	0	0	0	0	70	60	20	70
	Type I and II pneumocytes	0	0	0	0	0	0	0	0	4	4	3	4
	Exudate	0	0	0	0	0	0	0	0	1	0	1	1
	Bronchiolar epithelium	0	0	0	0	0	0	0	0	3	1	2	3

Table S2. Pathology and immunohistochemistry (IHC) scoring for transmission hamster samples. Hematoxylin and Eosin (H&E) stained samples were scored as follows: 0 = not present; 1 = 1-10%; 2 = 11-25%; 3 = 26-50%; 4 = 51-75%; 5 = 76-100%. IHC was scored as follows: 0 = not present; 1 = rare/few; 2 = scattered; 3 = moderate; 4 = numerous; 5 = diffuse. IN, intranasal; IM, intramuscular.

		IN-vaccinated animals				IM-vaccinated animals				Control animals			
H&E	Lesions %	0	0	0	0	0	20	10	5	50	50	50	40
	Interstitial pneumonia	0	0	0	0	0	3	3	2	3	3	3	3
	Bronchiolitis	0	0	0	0	0	0	0	0	3	2	2	2
	Alveolar exudate	0	0	0	0	0	2	1	2	2	3	2	2
	Type II pneumocyte hyperplasia	0	0	0	0	0	3	1	0	2	2	1	1
	Perivascular leukocyte infiltration	0	0	0	0	0	0	2	0	1	1	1	2
	Edema	0	0	0	0	0	0	0	0	3	1	2	2
IHC	Staining %	0	0	0	0	0	5	5	5	60	60	50	30
	Type I and II pneumocytes	0	0	0	0	0	1	1	1	4	4	4	2
	Exudate	0	0	0	0	0	2	0	0	1	1	1	1
	Bronchiolar epithelium	0	0	0	0	0	1	1	0	3	1	2	3
